# Cost-effectiveness for high dose quadrivalent versus the adjuvanted quadrivalent influenza vaccine in the Italian older adult population

**DOI:** 10.3389/fpubh.2023.1200116

**Published:** 2023-11-13

**Authors:** Filippo Rumi, Michele Basile, Americo Cicchetti, Fabián P. Alvarez, Maria Vittoria Azzi, Barbara Muzii

**Affiliations:** ^1^Alta Scuola di Economia e Management dei Sistemi Sanitari, Università Cattolica del Sacro Cuore, Rome, Italy; ^2^Sanofi, Lyon, France; ^3^Sanofi, Milan, Italy

**Keywords:** economic evaluation, cost-effectiveness analysis, influenza, vaccines, quadrivalent vaccines

## Abstract

**Objectives:**

To assess the cost-effectiveness of switching from adjuvanted quadrivalent vaccine (aQIV) to high-dose quadrivalent influenza vaccine (HD-QIV) in those aged ≥65 years from the Italian National Health Service perspective.

**Methods:**

We developed a decision tree model over a 1-year time-horizon to assess influenza-related costs and health outcomes. Two hospitalization approaches were considered: “hospitalization conditional on developing influenza” and “hospitalization possibly related to Influenza.” The first approach considered only hospitalizations with influenza ICD-9-CM diagnosis codes. The second included hospitalizations for cardiorespiratory events possibly related to influenza to better capture the “hidden burden”. Since comparative efficacy of high-dose quadrivalent influenza vaccine versus adjuvanted quadrivalent vaccine was lacking, we assumed relative efficacy versus a common comparator, standard-dose influenza quadrivalent vaccines (SD-QIV). We assumed the relative efficacy of HD-QIV vs. SD-QIV was 24.2 and 18.2% for the first and second hospitalization approaches, respectively, based on published information. Due to lack of comparative efficacy data for aQIV vs. SD-QIV, we assumed three different scenarios: 0, 6, and 12% relative efficacy in scenarios 1, 2, and 3, respectively.

**Results:**

For the first hospitalization approach, HD-QIV was a cost-effective alternative to aQIV in all scenarios at a willingness-to-pay threshold of €30,000 per Quality Adjusted Life Years. The incremental cost-effectiveness ratios across the scenarios were €7,301, €9,805, and €14,733, respectively, much lower than the willingness-to-pay per Quality Adjusted Life Years threshold. For the second hospitalization approach, HD-QIV was a dominant alternative to aQIV across all scenarios. The robustness of the results was confirmed in one-way and probabilistic sensitivity analyses.

**Conclusion:**

Switching to HD-QIV from aQIV for the older adult in Italy would improve health-related outcomes, and would be cost-effective or cost saving.

## 1. Introduction

Seasonal influenza is a recognized public health burden associated with substantial healthcare and economic costs. In Italy, surveillance data suggested that there were 8.7 and 8.1 million influenza cases in the 2017/18 and 2018/19 seasons (1, 2), respectively, with older adults, especially those with underlying chronic medical conditions, at a disproportionately higher risk of severe influenza-related outcomes and death (3, 4), despite much lower attack rates in general (3).

The influenza burden reported likely represents an under-reporting of cases, with a recent modelling study suggesting that only 18%–29% of influenza cases are detected through the Italian surveillance system (5). The magnitude of under-reporting may be compounded by the problem where the number of hospital admissions coded with an influenza diagnosis are likely much lower than the true number of influenza-related hospitalized cases (6). For example, there were an average 4,407 hospital admissions per year with the ICD-9-CM 487 code (related to the diagnosis of influenza) as the main diagnosis at discharge from the 2008–9 to 2014–5 influenza seasons. However, during the same time, hospitalizations that could be attributed in part to complications from influenza in the respiratory system (ICD-9-CM codes: 460–466, 481–486, 490–496, 500–508, and 510–516) and the circulatory system (ICD-9-CM codes: 422, 427, and 428) averaged (312,893 and 316,866) hospital admissions per year, respectively, suggest that the true influenza-related hospital burden may be more considerable. Of note, the length of stay of influenza hospitalizations (5.2 days) appears shorter compared to that associated with respiratory and circulatory diseases (up to 8.7 and 7.8 days, respectively, depending on disease severity) (6).

Annual vaccination has been central in the management of seasonal influenza, with the older adult (those aged ≥65 years) among the identified at-risk groups recommended for annual influenza vaccination (7, 8). However, the decline in immune response with age (immunosenescence) and subsequent reduced ability to respond to antigens may result in lower vaccine immunogenicity and efficacy in this vulnerable population (9). Thus, the development of more effective influenza vaccines for the older adult is an important medical need. in particular, the high-dose inactivated influenza quadrivalent vaccine (HD-QIV), containing four times the amount of antigen than standard-dose influenza quadrivalent vaccines (SD-QIVs), was developed to provide improved immunogenicity in older adults. Available data shows that high-dose inactivated influenza vaccines (both trivalent and quadrivalent) induce higher antibody responses than standard dose equivalents, and are more efficacious at protecting against influenza in the older adult (10–13). The MF-59 adjuvanted trivalent influenza vaccine (aTIV) was initially approved in Italy in 1997, and has been used in some Italian regions as the recommended vaccine for older adults, but is currently being replaced by the quadrivalent version (aQIV). However, there is a lack of robust randomized controlled efficacy data for aTIV or aQIV in older adults, and there is inconsistency in the results from observational studies (14).

To the best of our knowledge, there are no economic studies from the Italian National Health Service (NHS) perspective comparing HD-QIV with aQIV in the older adult. Here, we undertook a study to evaluate both health and cost outcomes of switching from aQIV to HD-QIV.

## 2. Methods

### 2.1. Description of the model

**Figure 1:**
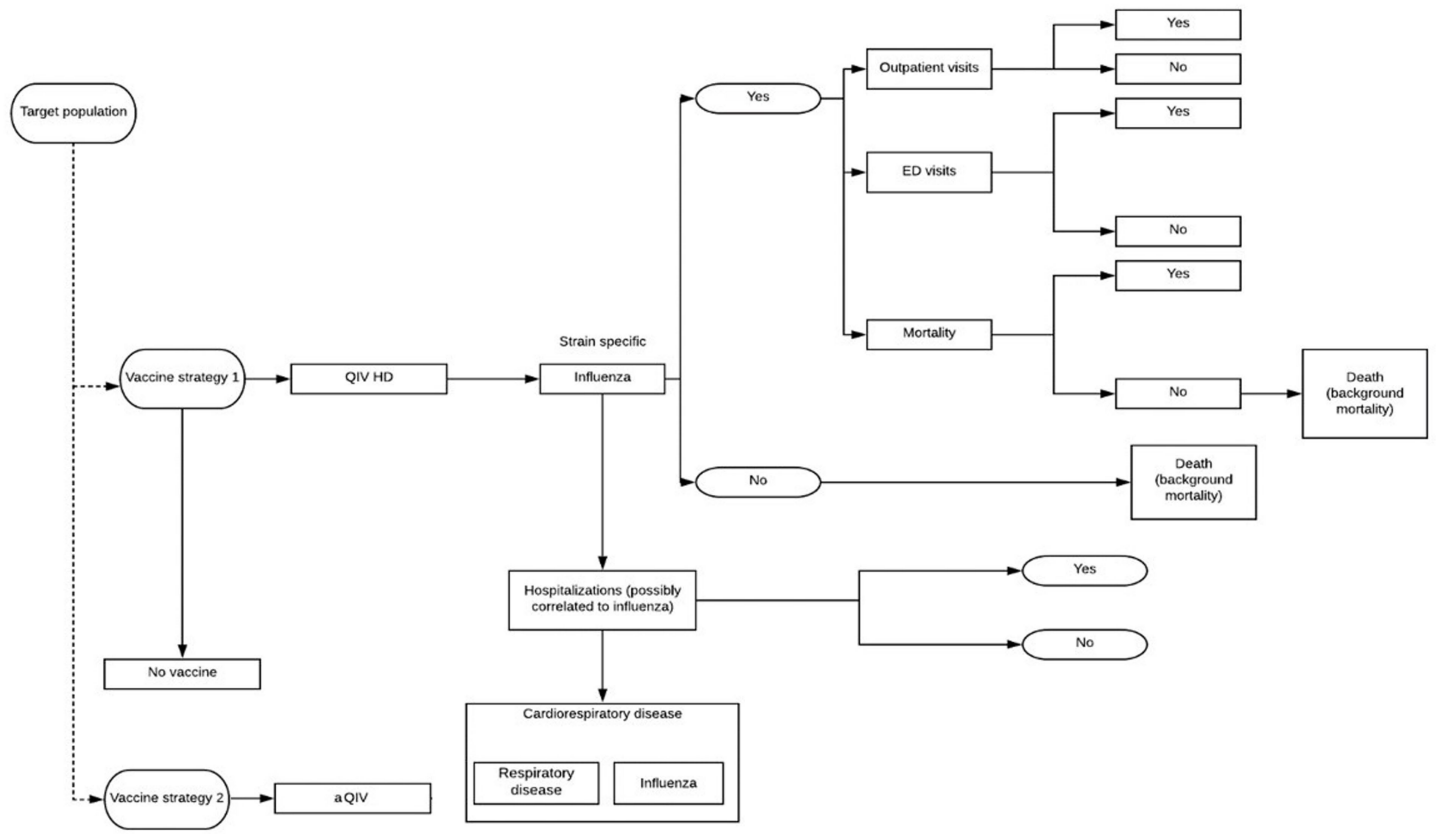
Decision tree model structure. Vaccine strategy 1: HD-QIV; vaccine strategy 2: aQIV.

We utilized a previously published decision tree model, described elsewhere in detail (15, 16), to compare the costs and benefits of switching from influenza vaccination with aQIV to HD-QIV in those aged ≥65 years from the Italian National Health System (NHS) perspective (Figure 1). The model estimates the health outcomes for both vaccines including general practitioner visits, emergency room visits, hospitalizations, deaths, and life years (Lys) and quality-adjusted life years (QALYs). A one-year time horizon was considered to account for all events of interest (health outcomes and costs) within a single influenza season and as such, discounting was not required. Long-term consequences were not considered with the exception of Lys and QALYs lost due to premature death which were considered over a lifetime horizon and discounted at 3.0% (15).

Two different hospitalization approaches were considered in the model described here. In brief, the first considered “hospitalization conditional on developing influenza”: this conservative approach requires the model to consider only hospitalizations associated with the diagnosis and coding of influenza. The second considered “hospitalization possibly related to influenza,” which included hospitalizations potentially attributable to influenza (i.e., respiratory and cardiorespiratory severe events) based on those documented by the investigators of the FIM12 randomized control efficacy trial (17). This later approach captures the ‘hidden burden’ related to a range of cardiorespiratory events that may be triggered by influenza, even though influenza was not diagnosed or codified (6, 10). The International Classification of Diseases 9 Clinical Modification (ICD-9-CM) codes included for each hospitalization definition are summarized in Supplementary Table S1. In order to estimate this burden we used the paper from Bertolani et al. (6). Authors ran a negative binomial model, in which the numbers of weekly admissions for respiratory and cardiovascular diseases were regressed based on Italian influenza syndrome surveillance data. On average over the 2008–2015 period, in addition to 4,407 admissions coded as influenza, authors of the study estimated 15,206 additional admissions attributable to influenza.

### 2.2. Model inputs

The model described here was adapted with Italian-specific population parameters where available including healthcare services and costs (Supplementary Table S2). The population considered is a hypothetical cohort of all those ≥65 years times the coverage rate, while the population of the budget impact model was estimated based on data from the National Institute of Statistics (18).

We assumed that the rate of general practitioner or emergency department services use by patients with laboratory-confirmed influenza was constant across all strategies and that both services could be used by patients (i.e., not mutually exclusive). Therefore, use of outpatient resources was directly linked to influenza attack rate and the respective efficacy against laboratory-confirmed influenza for each vaccine. The number of hospitalizations considered in the model was differentiated into the scenarios (HD-QIV and aQIV) based on the relative efficacy of the two hospitalization approaches. However, since there was no head-to-head clinical trial comparing HD-QIV and aQIV (neither their trivalent versions), the effectiveness of HD-QIV versus aQIV was calculated indirectly, based on the relative efficacy of each vaccine versus a common comparator, standard dose (SD-QIV).

The relative efficacy of HD-QIV vs. SD-QIV in the prevention of influenza cases was taken to be 24.2% as documented in the study by Diaz Granados et al. (17), which assessed the relative efficacy of HD-TIV vs. SD-TIV (FIM12 randomized controlled trial) in adults ≥65 years. The immune-bridging randomized controlled clinical trial, QHD00013 (19), supported the assumption that the same relative efficacy can be applied to HD-QIV vs. SD-QIV. The relative efficacy of HD-QIV vs. SD-QIV in preventing cardiorespiratory hospitalizations was considered to be 18.2%. Since there is no randomized controlled trial evidence for aQIV versus SD-QIV or for aTIV versus SD-TIV, there remains significant uncertainty regarding the relative efficacy of aQIV vs. SD-QIV, as concluded by the GRADE analyses carried out by the Canadian National Advisory Committee on Immunization (NACI) (20), European Centre for Disease Prevention and Control (ECDC) (14), and German Standing Committee on Vaccination (STIKO) (21). For both hospitalization approaches, in order to take into account these uncertainties, three different scenarios were investigated assuming relative efficacy of aQIV vs. SD-QIV of: 0% for scenario 1 (base case) (14, 20, 21), 6% for scenario 2 (22), and 12% for scenario 3 (23).

The average duration of influenza disease was assumed to be 6 days, and that for the duration of hospitalization possibly related to the influenza syndrome to be 5.2 days (to be conservative, length of stay for cardio-respiratory events was assumed to be equal to that for influenza) (6). The age stratified population mortality rate in Italy was retrieved from the Istituto Nazionale di Statistica (ISTAT) (18). The probability of death related to influenza was derived from Rosano et al. (3), considering vaccination coverage rate and the “excess mortality rate” per 100,000 inhabitants. As such, avoided deaths were not linked to hospital admissions but depended solely on the influenza cases avoided with each vaccine.

Cost data used in the economic model were obtained from Italian national databases and published literature (see Supplementary Table S2), and expressed in 2019 euros (€). Hospitalization cost was considered to be €4,035.32, accounting for the average hospitalization associated with Diagnosed-Related-Groups (DRGs) codes related to cardiorespiratory events (DRG 79, 80–81, 85–89). The aQIV and HD-QIV purchase costs used in the model were the Maximum Price to the Italian NHS.

Scalone et al. developed a study aimed to establish Health-Related Quality of Life (HRQoL) norm data for the general Italian adult population, allowing for comparisons with individuals affected by various diseases. A survey of 6,800 individuals, representative of Lombardy’s adult population in terms of age, gender, and geographical distribution, was conducted through telephone interviews. Their HRQoL was assessed using the EQ-5D-3L and EQ-5D-5L questionnaires, as well as the visual analogue scale (EQ-VAS), along with providing socio-demographic information. Italian population utility values were derived from the same study (24). In the QALY calculation, the model population expected life years accrued were adjusted by Italian population age-specific utility values (24). For patients with laboratory-confirmed influenza, the utility associated with clinically defined influenza was applied for the duration of illness (25), and those with influenza-related hospitalization, the utility associated with this outcome was applied (26). A willingness-to-pay threshold (WTP) of €30,000 per QALY was used to determine the cost-effectiveness of HD-QIV relative to aQIV. Incremental cost-effectiveness ratios (ICERs) lower than this WTP were considered cost effective.

### 2.3. Sensitivity analysis

Deterministic (one-way) and probabilistic sensitivity analyses were performed to assess the robustness of the results obtained in the base case (scenario 1) for the “hospitalization possibly related to influenza” outcome. The deterministic sensitivity analysis was let run for the three aQIV relative vaccine efficacy scenarios, with parameter ranges equal to 95% confidence intervals whenever available, or ± 15% of the mean if not. The probabilistic sensitivity analyses were performed over 1,000 simulations, with the assessed parameters and their respective distribution as described in Redondo et al. (16).

## 3. Results

### 3.1. Cost-effectiveness analysis

Table 1 summarizes the cost-effectiveness results for the first hospitalization approach, where “hospitalization conditional on developing influenza” was considered. HD-QIV was a cost-effective alternative to aQIV at a WTP of €30,000 per QALY, in all the scenarios explored for aQIV effectiveness.

**Table 1:** Summary of cost and benefits per individual aged >65 years in the population of HD-QIV versus aQIV: hospitalizations conditional on developing influenza.

	aQIV strategy	HD-QIV strategy	Incremental	ICER
HD-QIV vs. aQIV (where relative efficacy of aQIV vs. SD-QIV assumed as 0%)				
Total costs	15.44 €	24.11 €	8.67 €	–
Total LYs	10.2447	10.2460	0.00131	6,605 €
Total QALYs	8.8905	8.8917	0.00118	**7,301 €**
HD-QIV vs. aQIV (where relative efficacy of aQIV vs. SD-QIV assumed as 6%)				
Total costs	15.35 €	24.11 €	8.76 €	–
Total LYs	10.2450	10.2460	0.00099	8,870 €
Total QALYs	8.8908	8.8917	0.00089	**9,805 €**
HD-QIV vs. aQIV (where relative efficacy of aQIV vs. SD-QIV assumed as 12%)				
Total costs	15.27 €	24.11 €	8.84 €	–
Total LYs	10.2453	10.2460	0.00066	13,364 €
Total QALYs	8.8911	8.8917	0.00059	**14,733 €**

Table 2 and Supplementary Table S3 summarize the direct costs, clinical outcomes, and cost-effectiveness data for the second hospitalization approach, where “hospitalization possibly related to influenza” including cardiorespiratory events was considered, for the base-case scenario in which relative efficacy for aQIV was assumed to be null in comparison with the SD-QIV. The switch to HD-QIV could prevent 69,987 influenza cases, 27,015 influenza-related medical visits, 602 ED visits and 43,771 hospitalizations possibly related to influenza, thus saving the Italian NHS resources and associated costs amounting to €53.6 million, as well as improving clinical outcomes (i.e., LYs and QALYs gained). Thus, HD-QIV would be the dominant vaccination strategy in those aged ≥65 years in Italy. Of note, HD-QIV would save €176 million in hospitalizations costs due to cardiorespiratory events possible related to influenza.

**Table 2:** Summary of net population and per case clinical outcomes of HD-QIV versus aQIV (base-case results): hospitalizations possibly related to influenza.

Clinical outcomes	aQIV strategy	HD-QIV strategy	Differential	ICER
Net population clinical outcomes				
Cases of influenza	736,036	666,048	−69,987	
Influenza-related medical visits	284,110	257,095	−27,015	
Influenza-related emergency room visits	6,022	5,420	−602	
Hospitalizations – cardio-respiratory events	474,864	431,094	−43,771	
Deaths	6,129	4,309	−1,820	
QALYs	121,296,794	121,313,772	16,978	
LYs	139,782,102	139,800,015	17,913	
Per case clinical outcomes				
Total costs	153.82 €	149.9 €	−3.92 €	–
Total LYs	10.2447	10.246	0.0013	Dominant
Total QALYs	8.8899	8.8912	0.0012	Dominant

### 3.2. Sensitivity analysis

**Figure 2:**
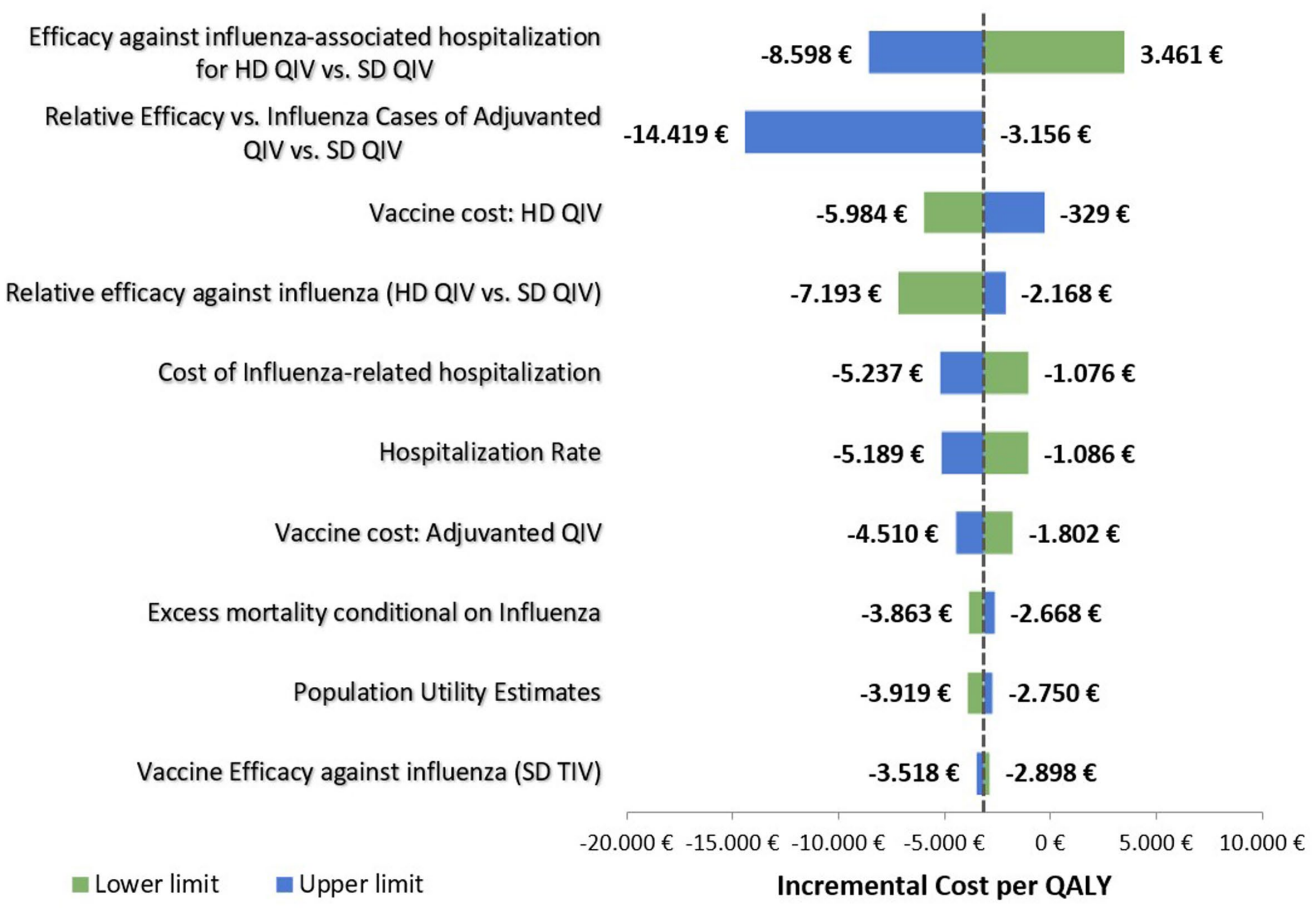
Deterministic sensitivity analysis: tornado diagram.

The deterministic (one-way) sensitivity analyses showed that efficacy against influenza-associated hospitalization for HD-QIV versus SD-QIV and the assumed relative efficacy against influenza for aQIV vs. SD-QIV had the greatest impact on the results (Figure 2). The most impactful parameter was the efficacy of the vaccination strategies to protect against influenza-related hospitalizations, as it can be seen in the Tornado plot (Figure 2). For increasing values of this parameter, the ICER obtained decreased up to −8.598 € (dominant strategy). On the other hand, when considering decreasing values of this parameter, the ICER (3.461 €) does not reach values above the reference threshold for cost-effectiveness (30.000 € per QALY). Also, the second parameter (relative efficacy vs. influenza cases of adjuvanted QIV vs. SD QIV) had an inverse correlation with respect to the cost-effectiveness ratio; an increase in the value of this parameter decreased the ICER.

**Figure 3:**
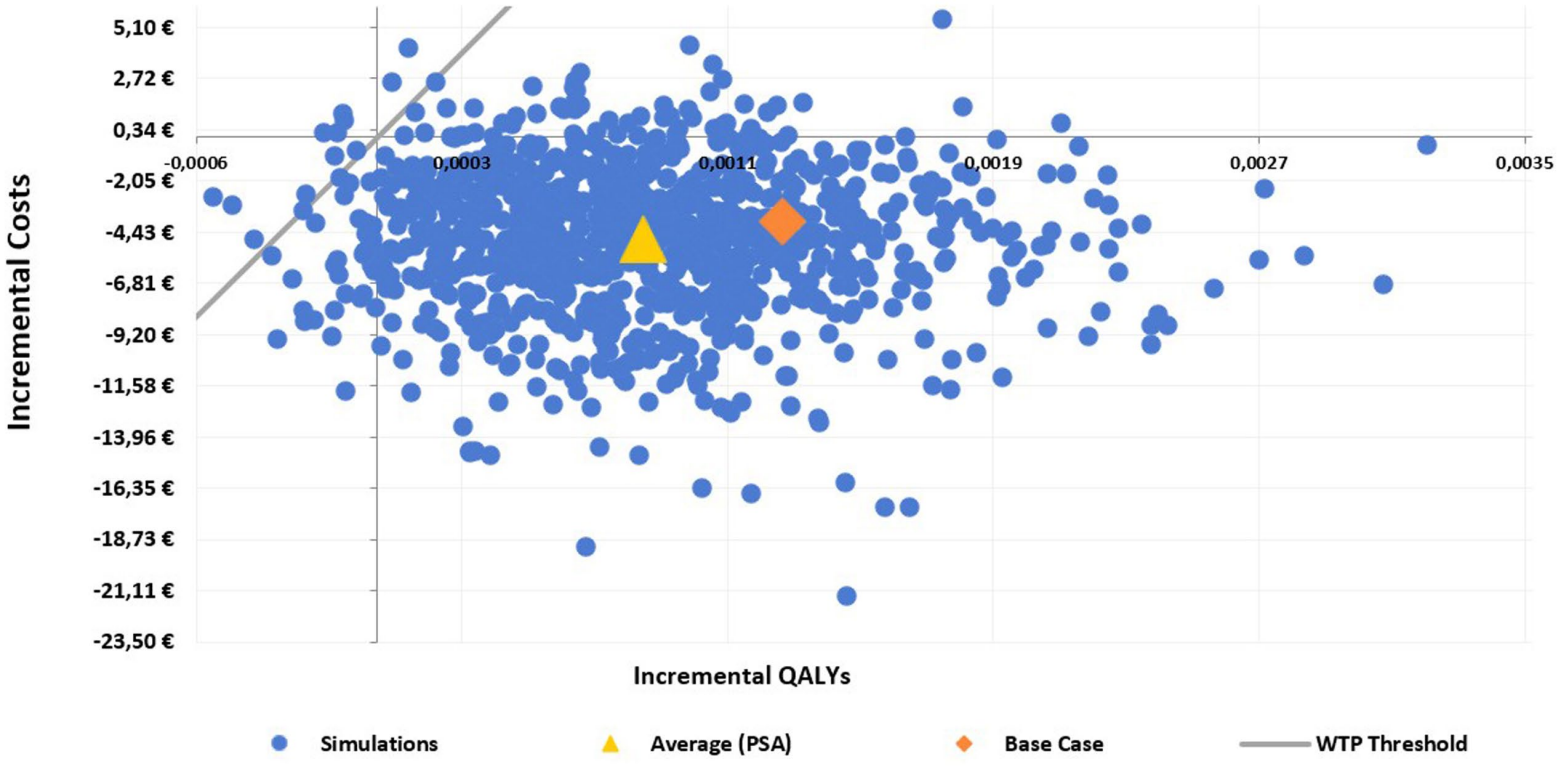
Probabilistic sensitivity analysis (PSA) scatterplot: cost-effectiveness plain for HD-QIV versus aQIV.

**Figure 4:**
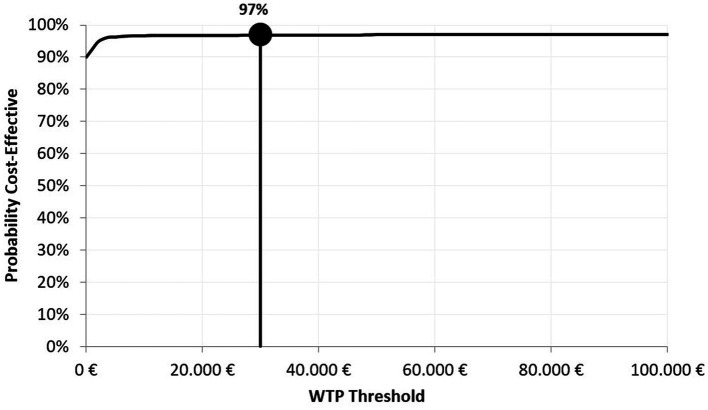
Cost-effectiveness acceptability curve for HD-QIV versus aQIV.

The incremental cost-effectiveness plane shows that HD-QIV is a dominant alternative to aQIV in the majority of the 1,000 simulations performed (i.e., most ICER values were located in the lower-right hand quadrant of the cost-effectiveness plane, indicating the lower incremental costs and higher QALYs with HD-QIV compared to aQIV) (Figure 3), and confirms the robustness of the results. The cost-effectiveness acceptability curve shows that HD-QIV had a high probability (97%) of being cost-effective compared with aQIV at a WTP threshold of €30,000 per QALY gained (Figure 4).

## 4. Discussion

The present study analyzed the cost-effectiveness profile of switching to HD-QIV from aQIV (standard of care) in the older adult from the perspective of the Italian NHS, considering both hospitalizations directly related and possibly related to influenza. Our study shows that switching to HD-QIV would result in improvement in health and related outcomes, as well as being at least cost-effective when considering a conservative approach in which only hospitalizations coded as influenza are taken into account. Moreover, when the potential hidden burden of influenza is considered by including hospitalizations potentially attributable to influenza (i.e., due to cardiorespiratory events), HD-QIV was a dominant option.

Several published studies have assessed the cost-effectiveness of high-dose vs. adjuvanted influenza vaccines in those aged ≥65 years, under different settings (15, 16, 27–29). Two studies assessed the cost-effectiveness of aQIV compared with HD-QIV (27, 28): the first – from the UK NHS perspective – suggested that aQIV (at a list price of £11.88) was cost-saving compared to HD-QIV, unless the later was priced lower than the existing list price of TIV-HD (£20.00) (27). The second, which estimated influenza-related costs and benefits in Spain over a one-year time horizon, suggested that aQIV would be cost saving from the medical payer and societal perspectives (28) engendered by an aQIV price almost half that for HD-QIV. Both studies assumed close effectiveness for HD-QIV and aQIV, based on a systematic review assessing observational data for aTIV/aQIV vs. SD-TIV and HD-TIV (30). However, that systematic review was criticized for not including all the available data (31), e.g., a failed randomized controlled trial of aQIV vs. a non-influenza vaccine (32).

Three other studies compared the cost effectiveness of HD-QIV versus aTIV in those aged ≥65 years in England and Wales, Spain, and Korea (15, 16, 29), from both the societal and healthcare perspectives. These studies suggested that HD-QIV would improve clinical benefits, while being at least cost-effective, if not the dominant option, when considering the burden possibly attributable to influenza (16, 29). Of note, the study undertaken England and Wales concluded that HD-TIV was a cost-effective alternative to aTIV in those aged ≥65 years from the healthcare perspective despite the price for HD-TIV being double that of aTIV (15). In our study, we utilized the maximum aQIV price to the Italian NHS (€15.45) and assumed a more than double price for HD-QIV (€32.27), and still showed that HD-QIV was a cost-effective alternative to aQIV, if not dominant, broadly consistent with the UK study. These economic assessments prioritized the use of the gold standard for efficacy measures, randomized clinical trials, and hence based the relative vaccine effectiveness inputs of HD-TIV efficacy from a randomized clinical trial (17) and a subsequent systematic review (observational and randomized clinical trial data) (10), acknowledged as grade A quality evidence supporting high-dose vaccines by the ECDC (33).

Our study has some limitation that should be considered when interpreting the results; these are discussed in detail in the previous publication utilizing the same decision tree model as in our study (16). First, since this is a static model, one of the limitation is that herd immunity effect may potentially be missed out and some cardiovascular related consequences from influenza occurring outside the time horizon considered are not fully accounted in the results. Furthermore, we chose not to take the social perspective into account because the target population analyzed was characterized by a low employment rate (we assumed most were retired), the inclusion of indirect costs (productivity losses) would not have significantly impacted the results. This, however, ignores productivity losses incurred by other adults involved in the care of the older adult. Thus, the benefits of the HD-QIV may not have been fully captured. We also assessed an outcome where “hospitalization possibly related to influenza” were included in our analysis. There is a lack of robust evidence regarding the proportion of cardiorespiratory hospitalizations that are truly related to influenza. Thus, we may have potentially overestimated or underestimated the benefits of HD-QIV with this later hospitalization outcome. Although we excluded out-of-pocket expenditure, the non-prescription drugs included in our model refer to in-hospital use incurred by the NHS, but which we assumed all influenza cases received, not only inpatients, thus slightly overestimating these costs in our analysis. Since our analysis, the relative efficacy of HD-QIV vs. SD-QIV in preventing hospitalizations for cardiorespiratory events possibly related to influenza was estimated as 17.9% (consistent with the 18.2% used in the current analysis) in a recent systematic review by Lee et al. (10). In addition, updated population parameters such as vaccination coverage have also been published the Ministry of Health of Italy. Nonetheless, these updates do not change our overall conclusion. Finally, the authors chose to conduct the deterministic sensitivity analysis solely on the base-case scenario, which focuses on hospitalizations potentially linked to influenza. This particular analysis exhibited the highest variability in results and, as a result, it was featured prominently in the paper. This approach was selected because it was deemed the most effective method for comprehensively evaluating the overall impact of influenza on the older adult population.

## 5. Conclusion

Switching to HD-QIV from aQIV appears a promising option for the older adult in Italy, and would result in improvement in health and related outcomes, and would be cost-effective or cost-saving from the NHS perspective.

## Précis

aQIV is recommended for protection against influenza in the older adult based on inconsistent efficacy whereas HD-QIV has robust efficacy data and switching would be cost-effective/cost-saving.

## Data availability statement

The original contributions presented in the study are included in the article/Supplementary material, further inquiries can be directed to the corresponding author.

## Author contributions

FR, MB, AC, FA, MA, and BM contributed to the conception and design of the study and interpretation of the data. MB and FR contributed to data acquisition and analysis. FR drafted the first version of the paper. MB, FA, and MA consolidated subsequent drafts. All authors contributed to the article and approved the submitted version.
