# Supplementary material for: Cost-effectiveness for high dose quadrivalent versus the adjuvanted quadrivalent influenza vaccine in the Italian older adult population
Source: Front Public Health. 2023 Nov 13;11:1200116. doi: 10.3389/fpubh.2023.1200116 (PMC10679352; doi:10.3389/fpubh.2023.1200116)
Supplement: Supplementary file 1 [file Table_1.DOCX]

**Supplementary material**

*Table S1 - ICD-9-CM codes and corresponding diagnoses*

| - ICD-9-CM codes | Diagnoses |
| --- | --- |
| - ICD-9-CM487 | - Influenza |
| - ICD-9-CM460–466 | - Acute nasopharyngitis - acute sinusitis, - acute pharyngitis, - acute tonsillitis, - acute laryngitis and tracheitis, - acute upper respiratory tract infections in multiple or unspecified locations, - acute bronchitis and bronchiolitis. |
| - 481–486 | - Pneumococcal pneumonia, - other bacterial pneumonia, pneumonia from other specified organisms, - pneumonia in infectious diseases classified elsewhere, bronchopneumonia, - unspecified agent, - pneumonia, unspecified agent. |
| - 490–496 | - Bronchitis, unspecified whether acute or chronic, - chronic bronchitis, - emphysema, - asthma, - bronchiectasis, - extrinsic allergic alveolitis, - chronic airway obstructions, not elsewhere classified |
| - 500–508 | - Coal workers pneumoconiosis, - asbestosis, other pneumoconiosis from silica and silicates, Pneumoconiosis from other inorganic dusts - Pneumoconiosis from inhalation of other dusts - Pneumoconiosis, unspecified - Respiratory morbid manifestations from inhalation of chemical fumes and vapors, - solid and liquid pneumonia, - Respiratory morbid manifestations from other and unspecified external agents |
| - 510–516 | - Empyema - Pleurisy - Pneumothorax - Abscess of the lung and mediastinum - Pulmonary congestion and hypostasis - Post-inflammatory pulmonary fibrosis - Other alveolar and parietoalveolar pulmonary diseases |

*Table S2. Summary of model input parameters and data sources used*

| Parameter | Input value | Source |
| --- | --- | --- |
| Demographics | | |
| Population aged ≥65 years | 13,644,363 | [23] |
| Background mortality (65–74 years) | 1.17% | [23] |
| Background mortality (≥75 years) | 4.20% | [23] |
| Influenza-related epidemiology | | |
| Immunization coverage among those aged ≥65 years | 54.6% | [35] |
| Attack rate among those aged ≥65years (unvaccinated) | 7.2% | [36] |
| Rate of access to the emergency room with influenza diagnosis (65–74 years) | 0.57% | [37] |
| Rate of access to the emergency room with influenza diagnosis (≥75 years) | 1.07% | [37] |
| Rate of visits to GPs with influenza diagnosis | 38.6% | [38] |
| Hospitalization rate per 100,000 for coded influenza (approach 1) | 75.4 | [6] |
| Hospitalization rate per 100,000 for influenza and cardio-respiratory events (approach 2) | 5,801 | [6] |
| % of hospitalizations during the influenza season | 60% | Assumption |
| Proportion of hospitalizations due to respiratory infections | 49.7% | [6] |
| Mortality rate conditional to influenza (65–74 years) | 2.58% | [3] |
| Mortality rate conditional to influenza (≥75 years) | 2.75% | [3] |
| Costs | | |
| aQIV vaccine | 15.45 € | Maximum Price to NHS |
| HD-QIV vaccine | 32.27 € | Maximum Price to NHS |
| Costs for using resources |  |  |
| Vaccine administration | 6.16 € | [39] |
| Prescribed influenza medicines | 20.78 € | [40] |
| Influenza non-prescribed medicines | 11.34 € | [41] |
| General practitioner visit related to influenza | 20.66 € | National tariff of specialist outpatient services |
| Access to the emergency room related to influenza | 261.35 € | Correction of commissioner decree N° U00265 September 1, 2014 |
| Hospitalization for influenza and cardio-respiratory events | 4,035.32 € | Data from Piemonte Region considering the following DRGs: 79, 80-81, 85-89, 92-93, 96-102, 565-566 |

*Table S3. Summary of global costs of HD-QIV versus aQIV (base-case results): hospitalizations possibly related to influenza*

| Costs | aQIV Strategy | HD-QIV Strategy | Differential |
| --- | --- | --- | --- |
| Vaccine | 114,922,686 € | 240,035,928 € | 125,113,242 € |
| Administration | 45,820,307 € | 45,820,307 € | 0 € |
| Prescription medicines | 5,903.800 € | 5,342,427 € | –561,373 € |
| Non-prescription medicines | 8,346,644 € | 7,552,988 € | –793,655 € |
| Influenza-related medical visits | 5,869,707 € | 5,311,576 € | –558,131 € |
| Influenza-related emergency room visits | 1,573,867 € | 1,416,451 € | –157,416 € |
| Hospitalizations | 1,916,229,411 € | 1,739,600,610 € | –176,628,800 € |
| Total | 2,098,666,423 € | 2,045,080,288 € | –53,586,135 € |

*Table S4. Summary of parameters used in deterministic sensitivity analysis*

|  | Inputs | | | ICERs | |
| --- | --- | --- | --- | --- | --- |
| Parameter | Base-case value | Lower Value | Upper Value | Lower limit | Upper limit |
| Efficacy against influenza-associated hospitalization for HD QIV vs. SD QIV | 18,2% | 6,8% | 28,1% | 3.461 € | -8.598 € |
| Relative Efficacy vs. Influenza Cases of Adjuvanted QIV vs. SD QIV* | 0% | 0% | 20% | -3.156 € | -14.419 € |
| Vaccine cost: HD QIV | 32,27 € | 25,82 € | 38,72 € | -5.984 € | -329 € |
| Relative efficacy against influenza (HD QIV vs. SD QIV) | 24,2% | 9,7% | 36,5% | -7.193 € | -2.168 € |
| Cost of Influenza-related hospitalization | 4.035,32 € | 3.228,26 € | 4.842,38 € | -1.076 € | -5.237 € |
| Hospitalization Rate (per 100.000) | 5.801 | 4.640 | 6.961 | -1.086 € | -5.189 € |
| Vaccine cost: Adjuvanted QIV | 15.45 € | 5,59 € | 8,39 € | - 1.802€ | - 4.510 € |
| Excess mortality conditional on Influenza (per 100.000) | 143,90 | 115,12 | 172,68 | -3.863 € | -2.668 € |
| Population Utility Estimates (mean) | 0,87 | 0,70 | 1,00 | -3.919 € | -2.750 € |
| Vaccine Efficacy against influenza (SD TIV) | 46,0% | 36,8% | 55,2% | -2.898 € | -3.518 € |
| % of total hospitalizations related to Respiratory causes | 49,7% | 39,8% | 59,6% | -3.324 € | -2.983 € |
| Mean influenza duration (days) | 6,00 | 4,00 | 12,00 | -3.189 € | -3.080 € |
| Utility per day of influenza-related hospitalization | 0,018 | 0,014 | 0,022 | -3.185 € | -3.128 € |
| Utility per day of influenza | 0,295 | 0.221 | 0.368 | -3.141 € | -3.172 € |
| Probability of death for the general population (mean) | 2,69% | 2,15% | 3,23% | -3.141 € | -3.171 € |
| Average duration of an influenza season (years) | 0,5 | 0,4 | 0,6 | -3.142 € | -3.171 € |
| % of a GP visit | 38,6% | 30,9% | 46,3% | -3.143 € | -3.169 € |
| Cost of Non-prescription influenza medications | 11,34 € | 9,07 € | 13,61 € | -3.147 € | -3.166 € |
| Cost of Prescription influenza medications | 20,78 € | 16,62 € | 24,94 € | -3.150 € | -3.163 € |
| Cost of Influenza-related GP visit | 20,66 € | 16,53 € | 24,79 € | -3.150 € | -3.163 € |
| % of a ED presentation | 0,8% | 0,66% | 0,98% | -3.154 € | -3.158 € |
| Cost of Influenza-related ED presentations | 261,35 € | 209,08 € | 313,62 € | -3.154 € | -3.158 € |
| Influenza Attack rate | 7,2% | 5,8% | 8,6% | -3.156 € | -3.156 € |
| Vaccine Administration Cost | 6,16 € | 4,93 € | 7,39 € | -3.156 € | -3.156 € |
| % of Influenza Cases by strain. | 70,2% | 56,2% | 43,8% | -3.156 € | -3.156 € |
| % of Mismatched B strain cases | 34,0% | 27,2% | 40,8% | -3.156 € | -3.156 € |
| Vaccine Coverage (Hospitalization Rate) | 54,4% | 54,4% | 54,4% | -3.156 € | -3.156 € |
| Efficacy against influenza-associated hospitalization for Adjuvanted QIV vs. SD QIV* | 0,00% | 0,00% | 20,0% | -3.156 € | -3.156 € |

*Three different scenarios will be developed considering a relative efficacy of 0%, 6% and 12%
